# Allicin, an Antioxidant and Neuroprotective Agent, Ameliorates Cognitive Impairment

**DOI:** 10.3390/antiox11010087

**Published:** 2021-12-30

**Authors:** Muhammad Shahid Nadeem, Imran Kazmi, Inam Ullah, Khushi Muhammad, Firoz Anwar

**Affiliations:** 1 Department of Biochemistry, Faculty of Science, King Abdulaziz University, Jeddah 21589, Saudi Arabia; anwarfiroz2000@gmail.com or; 2 Department of Biotechnology and Genetic Engineering, Hazara University, Mansehra 21300, Pakistan; drinamullah34@gmail.com (I.U.); khushi.muhammad@gmail.com (K.M.)

**Keywords:** garlic, allicin, biosynthesis, therapeutic, antioxidant, neuroprotective, cognitive impairment

## Abstract

Allicin (diallylthiosulfinate) is a defense molecule produced by cellular contents of garlic (*Allium sativum* L.). On tissue damage, the non-proteinogenic amino acid alliin (*S*-allylcysteine sulfoxide) is converted to allicin in an enzyme-mediated process catalysed by alliinase. Allicin is hydrophobic in nature, can efficiently cross the cellular membranes and behaves as a reactive sulfur species (RSS) inside the cells. It is physiologically active molecule with the ability to oxidise the thiol groups of glutathione and between cysteine residues in proteins. Allicin has shown anticancer, antimicrobial, antioxidant properties and also serves as an efficient therapeutic agent against cardiovascular diseases. In this context, the present review describes allicin as an antioxidant, and neuroprotective molecule that can ameliorate the cognitive abilities in case of neurodegenerative and neuropsychological disorders. As an antioxidant, allicin fights the reactive oxygen species (ROS) by downregulation of NOX (NADPH oxidizing) enzymes, it can directly interact to reduce the cellular levels of different types of ROS produced by a variety of peroxidases. Most of the neuroprotective actions of allicin are mediated via redox-dependent pathways. Allicin inhibits neuroinflammation by suppressing the ROS production, inhibition of TLR4/MyD88/NF-κB, P38 and JNK pathways. As an inhibitor of cholinesterase and (AChE) and butyrylcholinesterase (BuChE) it can be applied to manage the Alzheimer’s disease, helps to maintain the balance of neurotransmitters in case of autism spectrum disorder (ASD) and attention deficit hyperactive syndrome (ADHD). In case of acute traumatic spinal cord injury (SCI) allicin protects neuron damage by regulating inflammation, apoptosis and promoting the expression levels of Nrf2 (nuclear factor erythroid 2-related factor 2). Metal induced neurodegeneration can also be attenuated and cognitive abilities of patients suffering from neurological diseases can be ameliorates by allicin administration.

## 1. Introduction 

*Allium sativum* (garlic), a member of the Alliaceae family, is an essential component of human food since ancient times [1]. It is a rich source of vitamins, minerals, sulfur compounds, essential oils, phenols, and free amino acids [2]. Initial studies on the biochemical composition of garlic indicated the presence of my sulphur containing compounds especially, the polysulphides. Several bioactive compounds from garlic, including allicin, allyl sulphides, alliin, ajoenes, and 1,2-vinyldithiin have therapeutic effects as antioxidants, anti-inflammatory, cardioprotective, antimicrobial, anticancer, and immunomodulatory agents [3,4,5,6]. Allicin a sulphur containing bioactive compound, responsible for the typical fragrance of garlic was discovered in 1944, its chemical structure and mechanism of action against bacterial proliferation was studied [7]. Allicin is synthesized from a non-proteinaceous amino acid known as *S*-allyl-l-cysteine sulfoxide (alliin) that is hydrolysed by the enzyme alliinase [8]. Being a reactive sulphur species (RSS), allicin acts as an oxidizing agent in the cells and oxidizes thiols in the cysteine residues of proteins and glutathione [9]. 

Allicin has a wide spectrum of therapeutic applications. It has been used as an antimicrobial agent against many microorganisms such as *Staphylococcus aureus, Helicobacter pylori, Candida albicans,* and *Bacillus spp.* [9,10,11]. Allicin has inhibitory action against the activity of several enzymes by interaction the cysteine residues [9]. It is a health promoting compound that can reduce triglycerides and low-density cholesterol in the human body [12]. Allicin is an oxidant but at low concentrations and physiological conditions in the human body it promotes the production of antioxidant enzymes and inhibits the oxidation of low-density plasma lipids. It also inhibits the production of cholesterol in the human body and subsequently reducing the chances of blockage of arteries by plaque formation [13]. A low concentration of allicin (0.4 mM) can inhibit the platelet aggregation up to 90%, the impact is significantly higher than of similar concentration of aspirin. These properties suggest allicin as an efficient therapeutic agent against CVDs (cardiovascular diseases) [14]. Allicin decomposes rapidly and undergoes a series of reactions with glutathione resulting in the production of hydrogen sulphide (H_2_S). H_2_S is a gaseous signalling molecule involved in the regulation of blood pressure. It also regulates the relaxation of smooth muscles, dilation of arteries and lowering of blood pressure [15,16]. The downregulation of angiotensin II type 1 receptor and the NF-E2-related factor-2 (Nrf2)—inhibitor Keap1 has shown to facilitate the antihypertensive, antioxidant, and cardioprotective, activity of allicin [17]. 

**Figure 1:**
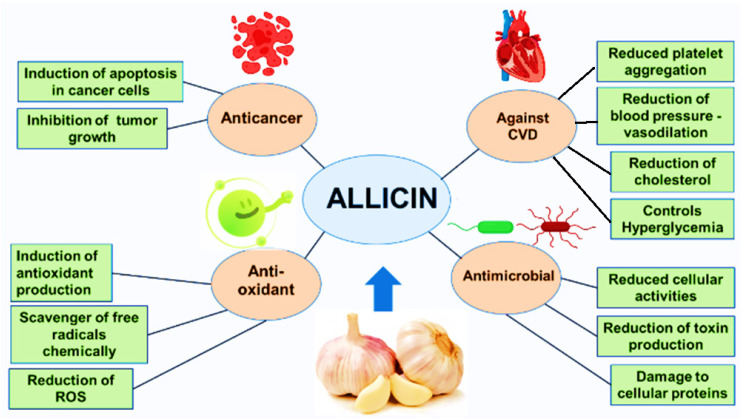
An illustration of general medicinal applications of allicin.

The anticancer activity of allicin involves a number of cellular mechanisms. It can change the redox status of cells resulting in the cell death [18]. By modulation of p53 pathway, allicin can promote cell cycle arrest and apoptosis in breast cancer cells [19]. With an inhibitory effect on ‘pain mediating molecules’ such as endothelin, IL-8 (interleukin 8), TNF α (tumour necrosis factor α), allicin can reduce the oral cancer pain [20]. Telomerase is an enzyme responsible for the addition of guanine-rich repeats to maintain the length of telomeres. The activity of enzyme is retained in stem cells and gametes. However, the telomerase activity is abolished after 50–70 cell divisions in the somatic human cells hampering any further cellular proliferations [21]. Allicin inhibits the activity of telomerase in a dose dependent manner subsequently inhibiting the proliferation in the cancer cells [22]. Preclinical studies have shown that allicin has positive impact on the healing of wound under diabetic conditions and streptozotocin-induced nephropathy in Wistar rats [23,24]. It has also shown the ability to alleviate hepatic toxicity induced by lead (Pb) and acrylamide [25,26]. Allicin has been reported to recover the spermatogenesis and sperm quality after diabetic induced damage, it has also shown beneficial effects on the reproductive system of male Wistar rats [27,28]. Diabetes mellitus is a complex metabolic disorder caused primarily due to the disturbed insulin release or insulin sensitivity [29]. Garlic is among the recommended neutraceuticals available on the market. Studies have shown a significant increase in the blood insulin levels after treatment with allicin [30] (Figure 1). 

In the nutshell, allicin is one of the most widely consumed neutraceuticals with multifaceted medicinal properties. It has an amazing potential in human health care and disease cure areas. In this context we aimed to evaluate the recent developments in the application of allicin as antioxidant and neuroprotective that helps to improve the patients with cognitive impairments. 

## 2. Biosynthesis, Properties and Action Mechanism of Allicin

Allicin is produced from *S*-allyl-L-cysteine sulfoxide (alliin)—a non-protein amino acid. The radiolabelling experiments have revealed that cysteine combines with glutamic acid to produce γ-glutamylcysteine, the later combines with glycine to prepare glutathione [31]. An odour producing substance known as alliin, or *S*-allyl-L-cysteine sulfoxide (ACSO) is one of the critically important constituents of the garlic bulb. Several contradictory pathways for the biosynthesis of alliin have been proposed and it has been a topic of debate [8,18,32]. Glutathione interacts with di-2-propenyl disulfide (DADS), the later undergoes a nucleophilic substitution reaction at α-carbon to produce allyl perthiol and *S*-allylglutathione (SAG). SAG is a potent antioxidant that has been evaluated to ameliorate the liver toxicity caused by carbon tetrachloride (CCl_4_) [33]. After losing glutamate and glycine, *S*-allylcysteine (SAC) is produced from *S*-allylglutathione, the reaction is catalysed by cysteinylglycinase and γ-glutamyl transpeptidase [34]. SAC is an antioxidant, anti-inflammatory compound that acts as a scavenger of ROS [35]. It is further converted to alliin [36], the latter has many important physiological properties. It helps to lower the hyperglycaemic conditions and improves the glutathione and catalase biosynthesis [37,38]. The alliin content varies according to the garlic varieties, in the dried garlic powder up to 1% alliin is found [4].

**Figure 2:**
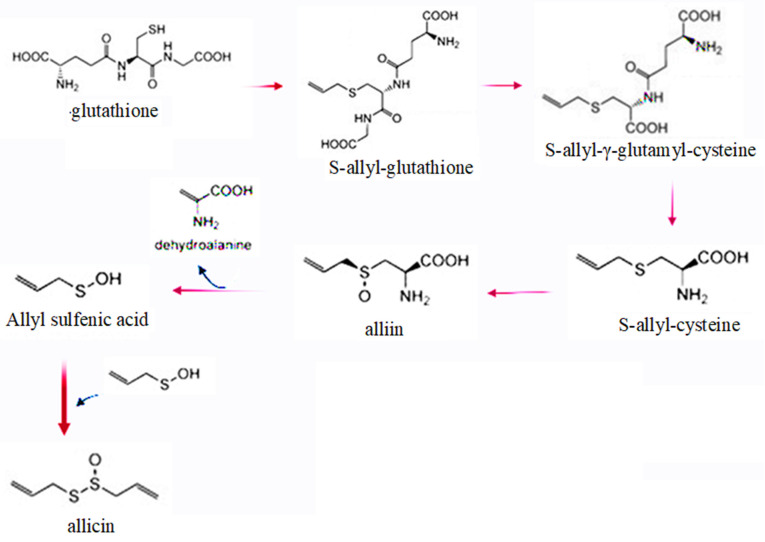
A schematic overview of allicin biosynthesis.

Application of alliin helps to promote glucose metabolism and insulin sensitivity [39,40]. Its applications have shown positive effects on the blood lipid profile and prevented heart attack [41]. Allicin (diallylthiosulfinate) is produced from alliin under the influence of enzyme alliinase (Figure 2).

**Figure 3:**
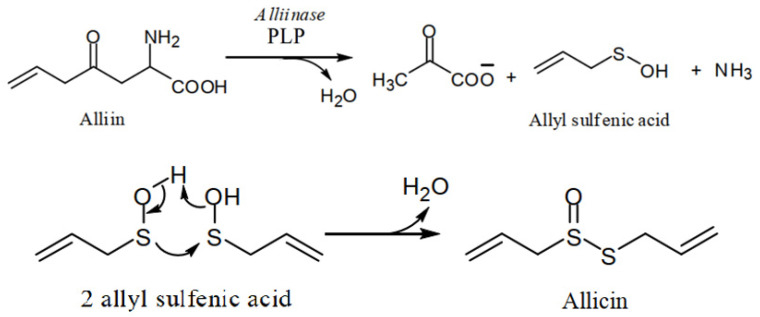
An elaborated scheme of allicin biosynthesis from alliin.

Allicin is not found in the intact garlic bulb, both the enzyme and alliin are found in different parts of garlic bulb. The reaction takes place when the garlic bulb is crushed. Enzyme and alliin make an enzyme-substrate complex in the presence of water. The dehydration is supported by pyridoxyl phosphate (PLP), the reaction results in the production of pyruvate, allyl sulfenic acid, and ammonia. The precursor alliin is found in four stereoisomers in nature, only one form ((+)-*S*-allyl-l-cysteine-sulfoxide) is found in the garlic. The process of allicin production is associated with the defence mechanism of garlic plant. After invasions to the garlic clove cells, enzyme and alliin are released and allicin is produced immediately to destroy the invader. At room temperature, allyl sulfenic acid is condensed in to allicin, optimum temperature for the activity of alliinase is 33 °C, it operates best at pH 6.5, the enzyme is sensitive to acids [42,43] (Figure 3), enteric-coated formulations of garlic supplements are therefore recommended [44].

**Figure 4:**
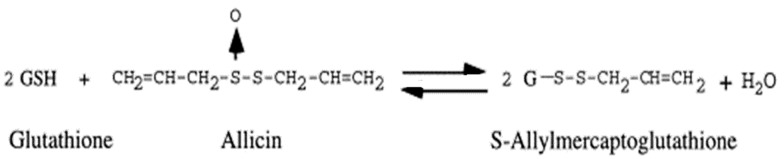
Reaction of GSH with allicin.

Allicin, one of the 30 sulphur containing compounds of garlic volatile and short-lived substance. Its concentration in the human plasma, urine and stool samples cannot be determined accurately due to instability. It has molecular weight of 162.28 g/mol and water solubility up to 2.40 × 104 mg/L [45]. It can readily cross the cellular membranes due to its hydrophobic nature and reacts with thiols [46,47]. For the general metabolic reactions, it is obligatory to maintain the cellular pH, ionic concentration and redox potential. The concept of redox potential comes from thermodynamics, it decides the possibility, direction and equilibrium point of a cellular reaction [48,49]. Under the normal conditions, the healthy cells have a negative redox potential. As for example in case of *Saccharomyces cerevisiae* the redox potential is from −220 mV to −320 mV [50,51]. Redox potential in the cells is regulated by the ratio of NADPH/NADP^+^ (coenzyme pool), ratio of GSH/GSSG (reduced glutathione/oxidized glutathione), and thioredoxins [52]. Allicin has oxidizing properties, oxidizes thiols in the cysteine residues found in the structure of proteins and in glutathione (Figure 4). 

Oxidation of proteins may result in the loss/gain of protein function due to the alterations in structures, changes in cellular physiology, and more oxidized glutathione leads to higher cellular redox potential [8]. Excessive generation of ROS is one of the basic factors responsible for insulin resistance resulting in diabetes and related metabolic disorders [53,54]. Allicin is found to inhibit the generation of ROS and subsequent insulin resistance [55,56]. ROS production is also promoted by hyperglycaemia that leads to myocardial apoptosis [57]. The mechanism of apoptosis is regulated by the balance between pro-survival and pro-apoptotic factors. One of the pro-survival factor Bcl2 mainly decides the fate of cells. According to recent findings, allicin can significantly reduce the expression level of Bcl12 in diabetic rats resulting in the reversal of myocardial apoptosis [58]. 

## 3. Allicin as an Antioxidant

**Figure 5:**
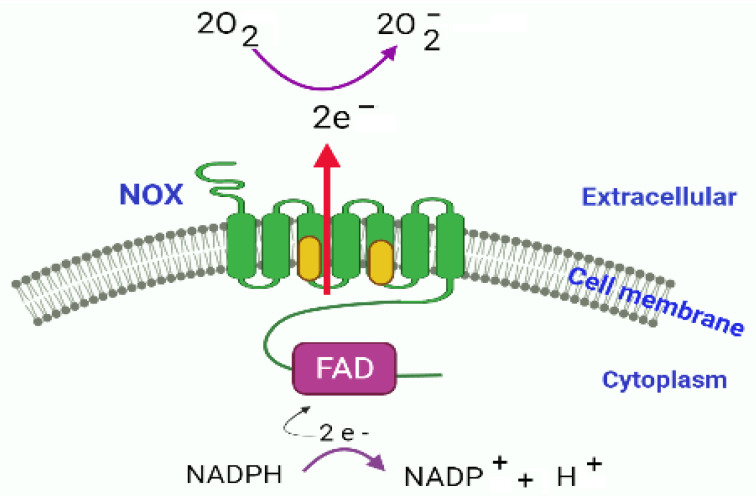
NOX electron transport system comprising of NOX transmembrane domains associated with heme molecules (indicated as yellow) at domain 3 and 5, C terminal region containing FAD and NADPH binding sites, transfer of electrons is catalysed by NOX. The transport of electrons results in the production of superoxide ion O_2_^−^.

Natural products are considered as better therapeutic agents against oxidative stress due to their minimum adverse effects [59]. Allicin has been reported as an antioxidant natural product. The antioxidant properties of allicin can be described on the basis of its ability to inhibit superoxide, nitric oxide (NO) and hydroxyl radicals [60,61]. ROS are highly unstable molecules that create oxidative stress when accumulated in the cells and cause tissue damage [62]. There are enzymic reactions that promote cellular oxidative stress, as for example, nicotinamide adenine dinucleotide phosphate oxidases (NOXs) are a series of complex enzymes considered as one of the major sources to produce reactive oxygen species resulting in inflammation and oxidative stress [63,64,65]. Each enzyme of NOX series is composed of six transmembrane domains and have conserved sites for the binding with FAD and NADPH (except NOX_5_). There are also haem-binding sites in the third and fifth transmembrane domains associated with electron transporter using NADPH from cytoplasm as the electron donor. The transported electrons are accepted by oxygen in the extracellular environment (Figure 5).

ROS species generated by NOX based system mainly include H_2_O_2_/NO [66], NOX2 and NOX4 contribute maximum ROS, and the expression level of these enzymes was significantly reduced by the treatment with allicin [67]. In the presence of redox-active ions such as Fe^2+^, the hydroxyl radical (•OH) can be produced by H_2_O_2_. Several other types of oxidants can also be produced by the action of peroxidases, as for example, generation of HOCl (hypochlorous acid) in the neutrophils by the action of myeloperoxidase (MPO). The nature of oxidants also depends on the type of cellular scavenger enzymes such as catalase (CAT), superoxide dismutase (SOD), and glutathione peroxidase (GPx) [68]. In addition to have a suppressive role in the ROS production by the inhibition of NOX enzyme systems, allicin also promotes the detoxifying enzymes [69,70]. According to another report, allicin has protective effect against H_2_O_2_ induced apoptosis in the human umbilical vein endothelial cells (HUVECs) [71,72]. Allicin can modify the levels of phase II detoxification enzymes such as heme oxygenase 1, (HO-1) thioredoxin reductase 1 and glutamate L-cysteine ligase (Glcl) [73,74]. SAMG (*S*-allylmercaptoglutathione) and SAMC (*S*-allylmercaptocysteine) are the products of allicin with GSH and cysteine respectively. SAMG is considered a powerful antioxidant derivative of allicin [75]. In the human eyes, the RFEs (retinal pigmented epithelial cells) make a layer of epithelial cells with high metabolic activity and ROS rensitivity [76]. On exposure to ROS, the damaged RPEs contribute to the pathogenesis of irreversible blindness, known as age-related macular degeneration (AMD) [77]. Excessive ROS production especially H_2_O_2_, or imbalanced homeostasis of ROS have been reported as the main risk factors of AMD [78,79]. According to the reports allicin plays an important role in the regulation of H_2_O_2_ and protects RPEs damage [80].

**Figure 6:**
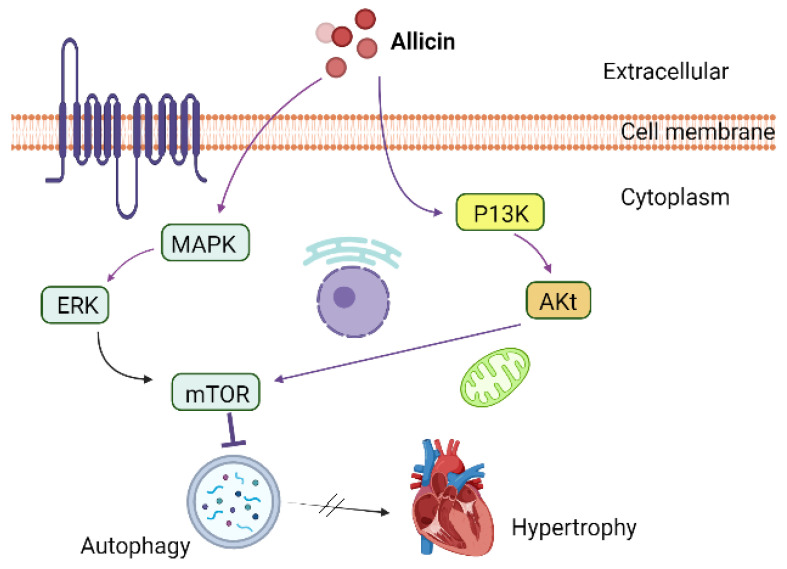
Allicin attenuated cardiac hypertrophy by regulation of autophagy via mTOR regulatory pathways.

Cardiac hypertrophy is the main cardiovascular concern worldwide [81]. It can lead to cardiac arrest, cardiac dysfunction, and sudden cardiac death [82]. Autophagy is the second type of programmed cell death responsible to get rid of aged-exhausted proteins and cellular organelles [83]. However, under certain circumstances such as in cancerous conditions, autophagy can involve in cellular remodelling [84,85]. Autophagy also plays an important role in the onset of cardiac diseases such as cardiac hypertrophy. According many recent reports, suppression of ROS, inflammation and autophagy can attenuate cardiac hypertrophy [86,87]. Allicin has been reported for its critical role in the hyperlipidaemia, cardiac failure and myocardial infarction [88]. It has also been reported to attenuate cardiac hypertrophy via regulation of ROS-dependent signalling pathways, and Nrf2 antioxidant signalling pathways, and activating PI3K/Akt/mTOR and MAPK/ERK/mTOR pathways (Figure 6) [89].

## 4. Allicin as a Neuroprotective Agent to Fight against Neurological Diseases

In the recent times, allicin has been extensively investigated as a neuroprotective agent [8,18,90,91]. Pathophysiology of several neuropsychological, neurological diseases, neurodegenerative diseases, and neurological damages such as spinal cord injury, traumatic brain injury, stroke, and neurotoxicity are accompanied by neuroinflammation [92,93]. Neurons are cells with high metabolic rates and essentially require abundant and efficiently working mitochondria. Therefore, the mitochondrial dysfunction is mostly associated with the pathogenesis of neurological conditions [94,95,96,97,98]. The causes for the neurological diseases also include deleterious mitochondrial DNA [99], accumulated misfolded proteins [100], problems in the calcium influx [101], flaws in the mitochondrial oxidative phosphorylation systems (OXPHOS) [102], elevated levels of ROS [103], and apoptosis of neuron cells [104]. After the cellular death, released DNA, proteins and cellular debris promote microglia, initiate inflammation and damage the tissue [95]. Hence, mitochondria play a critical role in the onset of neuroinflammation and its subsequent pathological events [105,106]. Microglia are the macrophages responsible for response to tissue damage and repair in the brain [107]. The activation of microglial cells participates in neuroinflammation and neurodegenerative diseases [108]. The activated microglia migrate, proliferate, releasing pro-inflammatory cytokines such as interleukin 1 beta (IL-1β) [109], tumor necrosis factor alpha (TNF-α) [110]. Some neurotoxic substances are also released leading to the death or dysfunction of neurons [111]. 

Several signalling pathways leading to neuroinflammation have been described [93]. The toll like receptors (TLRs) are important components of immune system that recognize foreign ligands and induce inflammation by the activation of corresponding signaling molecules [112,113]. A range of TLRs are expressed by microglia for their activation and initiation of neuroinflammation [114]. The binding of cytoplasmic domain of TLR with Myeloid Differentiating factor 88 (MyD88) leads in the activation of NF-κB (nuclear factor-kappa B), TLR4/MyD88/NF-κB signalling pathway promotes inflammation [115,116,117,118]. Inflammation causes DNA damage by the induction of oxidative stress, produces ROS in the microglia and promote aging process [119,120]. ROS can trigger the expression of many proinflammatory genes and further promote inflammation [121,122]. The reactions catalysed by two isoforms of cyclooxygenases, (COX1) and (COX-II) are also associated with neuroinflammation. Both of the isozymes catalyse dioxygenation of arachidonic acid and produce prostaglandin G2 (PGG2) which is further converted into prostaglandin H2 (PGH2) by the action of a peroxidase. A neuroinflammatory mediator PGE2is produced from PGH2 [123,124]. COX II has more prominent role in the induction of neuroinflammation and COX I is generally considered as the house keeping enzyme [125]. 

The signalling molecules of PI3K/AKT pathways are induced by the activation of microglia that initiate neuroinflammation [126,127]. Mammalian target of rapamycin (mTOR) is a typical serine/threonine kinase, a typical member of PI3K related family of kinases. Phosphorylation/activation of mTOR by the activation of p13K and AKt regulates the activity of NF-κB and results in neuroinflammation [128,129]. Activation of microglia also induces the activation of mitogen-activated protein kinase (MAPK) family kinases such as p38 MAPK and stress-activated protein kinases/Jun amino-terminal kinases (SAPK/JNK). P38 MAPK activates the production of proinflammatory cytokines and SAPK/JNK promote the expression of several genes associated with inflammation [130]. 

**Figure 7:**
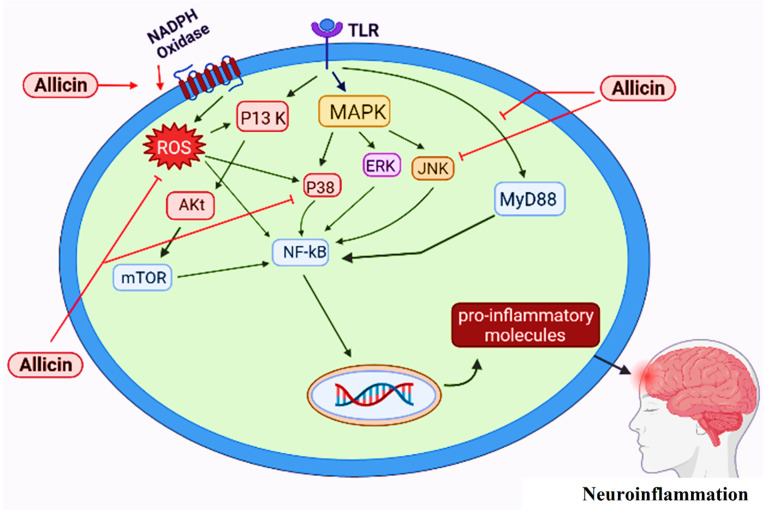
Proposed protective role of allicin against neuroinflammation based on recent literature.

Activation of microglia and pathways leading towards neuroinflammatory process have been described by several studies. Activation of microglia by the ligand binding at TLRs leads to the activation of the MAPK pathway. By downstream activation of P38 and/or JNK it activates NF-kB which regulates the production of proinflammatory cytokines. Allicin inhibits/suppresses P38 and JNK pathways and attenuates the production of pro-inflammatory molecules resulting in the anti-inflammatory response [131]. TLRs initiate another pathway known as TLR4/MyD88/NF-κB signal transduction pathway [132,133], which also leads to NF-kB induced production of proinflammatory molecules. Allicin has the ability to inhibit the TLR4/MyD88/NF-κB pathway consequently reducing the production of pro-inflammatory cytokines and inactivating the inflammatory machinery [134]. NADPH oxidases are membrane associated enzymes (with mode of action already described Figure 5). The activity of NOX results in the overproduction of ROS [131,135]. ROS can promote the production of proinflammatory molecules by activating NF-kB either directly or indirectly via P13K/AKt/mTOR/NF-kB pathway. Application of allicin has found to reduce the expression level of ROS generating enzymes (NOXs) decreasing the ROS in the cells [67]. In this way, allicin protects against neuroinflammation by interacting at various molecular and signalling transduction levels (Figure 7).

Alzheimer’s disease (AD) is a neurodegenerative condition, typically characterized in old-aged people. It is the most common cause of dementia, memory loss, depression and language impairments [136]. The main causes of AD include accumulation of amyloid β (Aβ) in the form of plaques [137], or deposition of Tau protein in the neurological tissues [138]. Declined levels of neurotransmitter acetylcholine (Ach) by the action of acetylcholinesterase (AChE) and butyrylcholinesterase (BuChE) also result in the onset of AD. The disease symptoms are mostly treated by the inhibition of these two enzymes [139,140,141,142]. Allicin has shown an inhibitory effect on the activity of AChE/BuChE enzymes. Application of allicin slows down the death of neurons and reduced the impaired cognitive functions in AD [143,144,145]. The level of Tau protein was reduced significantly by the use of allicin [146]. 

Acute traumatic spinal cord injury (SCI) characterized by the ischemia, bleeding, oxidative stress, neuronal inflammation, nerve degeneration and apoptosis [147,148,149,150]. In general, the activation of erythroid 2-related factor 2 (Nrf2)/antioxidant response element (ARE) pathway is most important mechanism against oxidative stress [151,152,153]. Preclinical studies on rabbits have shown protective effects of allicin against spinal cord reperfusion injury [154]. Allicin has been reported to protect SCI induced neuron damage by regulating inflammation and apoptosis and promoting the expression levels of Nrf2. No effect of allicin was observed in the Nrf2 knockout animals indicating that the effect of allicin involves Nrf2/ARE pathway [155]. In case of traumic spinal cord injury (TSCI), allicin can reduce the ROS levels and enhance NADPH levels by regulation of HSP70, Akt and iNOS pathways [156,157]. Toxic effects of acrylamide (ACR) on the peripheral and central nervous system is well established [158,159]. The combined therapy with allicin and melatonin has shown recovery of ACR damaged neurons by regulating DNA damage, increasing the levels of neurotransmitters [160]. 

**Figure 8:**
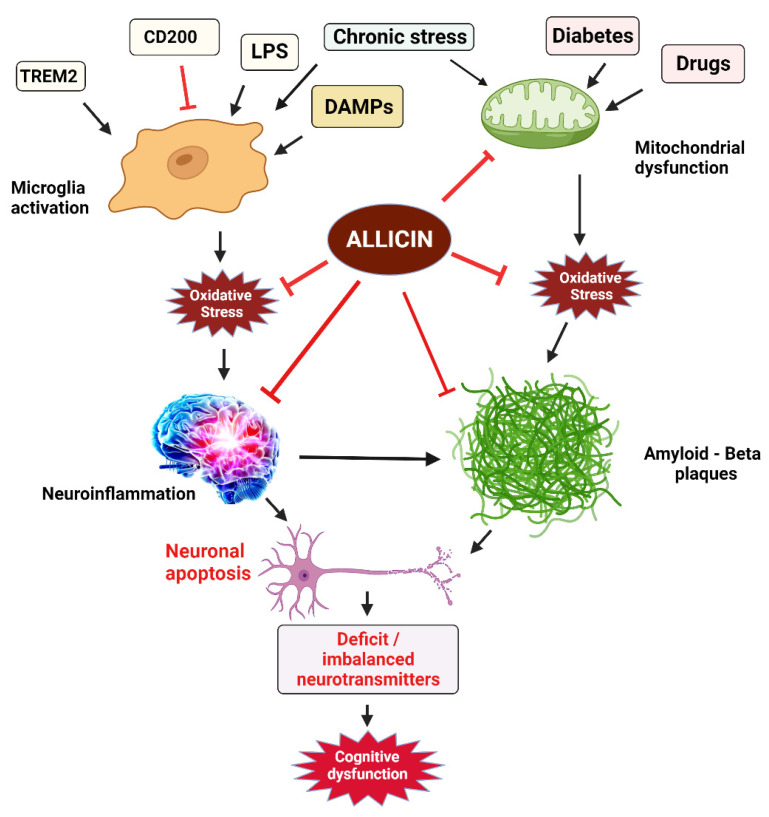
Role of allicin to ameliorate cognitive impairment adopted from various reports available in the literature.

Cognitive functions include multiple mental abilities such as remembering, decision making, thinking, problem solving, learning, reasoning, and attention towards surrounding activities. Impaired cognitive functions are often result of neurodegenerative conditions such as AD, and Parkinson’s disease (PD) [161,162,163,164]. Some other neuropathological conditions can also be represented by cognitive impairment such as autism spectrum disorder (ASD) [165,166,167], and attention deficit hyperactive disorder (ADHD) [168,169]. The cases of cognitive impairments result in the lower quality of life among the suffering individuals, increase social and economic burden to the society in general and to the families of patients in particular [170,171,172,173,174]. Imbalanced levels of neurotransmitters such as glutamate, dopamine, acetylcholine, and GABA are linked with cognitive impairments [175]. Exposure to higher metal induced oxidative stress, neurotoxicity and neurodegeneration can also cause cognitive deficits [176,177,178]. In the recent years several studies have reported the improvement in the cognitive skills of suffering individuals by the use of allicin. As for example, in a preclinical study, administration of copper and aluminium resulted and elevated levels of pro-inflammatory cytokines, oxidative stress, and altered levels of neurotransmitters. Allicin has shown antioxidant activity, restored the levels of neurotransmitters and reduced the inflammatory cytokines [179]. The cell membrane of microglia has receptors for the recognition scavenger, cytokines and chemokines and cells are activated by binding of any of these molecules to the corresponding receptors [180]. Activation and resting state of microglia are regulated by a set of molecules. As for example CD200 is a molecule produced on neurons and its corresponding receptor is CD200R on microglia. Binding of CD200 with its receptor inhibits the activation of microglia and retains its resting state [181]. The other common ligands that keep the resting state of microglia include CD172a/Sirp alpha, CD200R, and CX3CR1. TREM2 (triggering receptor expressed on myeloid cells 2) mimic the neuronal injury and activates the microglia [182]. The balanced regulation of cell surface ligands and receptors is necessary for the homeostasis of microglia, any dysregulation in this system may lead to adverse changes in microglia that can be damaging to the neuronal networks, leading to neuropathological events in adults and developmental issues in the young [183,184]. Activation of microglia is also regulated by lipopolysaccharides (LPS) and damage-associated molecular patterns (DAMPS) which leads to the ROS production, neuroinflammation or nerve damage [185]. Microglia can be activated by chronic psychological stress can have several downstream consequences including neurobiological complications and mental illness [186,187]. In addition to that mitochondrial dysfunction induced by some drugs [188,189], diabetes [190], and other factors, has been well associated with the onset of neurodegenerative diseases. Administration of allicin attenuates oxidative stress, mitochondrial dysfunction, apoptosis, inhibits neuroinflammation [191,192]. Hence, allicin improves cognitive ability by attenuating the upstream oxidative stress, mitochondrial dysfunction and inflammation. A proposed mechanism of elevation of cognitive functions by the application of allicin is summarized (Figure 8).

## 5. Limitations

Allicin has been extensively studied and reported for its therapeutic potential as an antioxidant with antimicrobial, anticancer, and anti-inflammatory activities. There are numerous reports on the neuroprotection and improvement of cognitive abilities by the application of allicin. However, almost all study reports on these subject areas are based on preclinical studies conducted on animal models or human cell lines and only two or three specific clinical studies have been reported. Crushed garlic material mainly consisting of allicin has been reported to treat thrush (whitish patches of yeast infection that cover the mouth) in newborn infants [193]. In a small-scale clinical study involving only 20 patients, allicin has been reported to treat Behcet’s disease [194]. In a randomized double blind placebo trial involving 96 patients, allicin tablets were effectively used for the treatment of aphthous ulceration with no significant side effects [195]. In another study on 52 Chinese patients (six male and 46 females) suffering from stage II oral submucous fibrosis (OSF), allicin was injected for 16 weeks intralesionally which gave significant improvement [196]. Only one clinical trial has been reported in clinicaltrials.gov where allicin is being applied for the treatment of cancer, no results have been yet reported. Allicin was also found effective against common cold in a trial [197]. Human trials and dose optimization studies are required for the establishment of allicin as a neuroprotective agent and in the improvement of cognitive functions.

## 6. Conclusions

Allicin is a volatile substance produced from amino acids by enzyme catalysed reactions in the crushed garlic cloves. Its potential as a potent antioxidant have been recognized in the management of pathogenic microbes, cancers and CVDs. Administration of allicin can reduce the ROS by reducing the expression of ROS producing NOX enzymes and promoting the CAT, SOD, GPX and several types of peroxidases. Allicin has been found a useful natural compound against neuroinflammation, in the management of neurodegenerative diseases such as AD, PD and psychneurological conditions including ASD, and ADHD. It can protect the neurons and nervous system, improves the cognitive abilities of patients suffering from neurological diseases. However, clinical studies are required to establish the therapeutic efficacy of allicin.
